# Late recurrence of breast cancer is associated with pro-cancerous immune microenvironment in the primary tumor

**DOI:** 10.1038/s41598-019-53482-x

**Published:** 2019-11-15

**Authors:** Takashi Takeshita, Li Yan, Mariko Asaoka, Omar Rashid, Kazuaki Takabe

**Affiliations:** 1 Breast Surgery, Department of Surgical Oncology, Roswell Park Comprehensive Cancer Center, Buffalo, NY USA; 2 Department of Biostatistics and Bioinformatics, Roswell Park Comprehensive Cancer Center, Buffalo, NY USA; 3 Holy Cross Hospital, Trinity Health, Fort Lauderdale, FL USA; 4 Massachusetts General Hospital, Boston, MA USA; 5 University of Miami Miller School of Medicine, Miami, FL USA; 6 Department of Surgery, University at Buffalo Jacobs School of Medicine and Biomedical Sciences, the State University of New York, Buffalo, NY USA; 7 Department of Breast Surgery and Oncology, Tokyo Medical University, Tokyo, Japan; 8 Department of Surgery, Yokohama City University, Yokohama, Japan; 9 Department of Surgery, Niigata University Graduate School of Medical and Dental Sciences, Niigata, Japan; 10 Department of Breast Surgery, Fukushima Medical University, Fukushima, Japan

**Keywords:** Breast cancer, Cancer genomics

## Abstract

The fact that 20–40% of all breast cancer (BC) patients develop recurrence when 5 year survival is 90% strongly suggests that late recurrence, i.e. more than 5 years after diagnosis, is the remaining challenge to decrease the absolute number of BC deaths. Better understanding late recurrence is an essential first step to address this issue. We hypothesized that primary tumors with a distinctive tumor immune microenvironment will develop late recurrence. Accordingly, we evaluated the relationship between the timing of cancer recurrence, clinical factors, gene expression profiles, and immune status utilizing two published large cohorts. 308 primary BCs in TCGA were analyzed and categorized as: recurrence ≤2 years (Early, n = 49), between 2–5 years (Mid, n = 54), recurrence >5 years (Late, n = 20), and no recurrence >5 years (Survivors, n = 185). 1,727 primary BCs in METABRIC were analyzed and categorized similarly: Early, n = 170; distant (D), n = 19; local (L), Mid, n = 213; D, n = 21; L, Late, n = 199; D, n = 57, L, and Survivors, n = 1048. Utilizing pre-ranked GSEA, we showed that primary tumors with Survivors were associated with anti-cancer signaling such as INF-α/-γ response and TNF-α signaling, compared with all recurrence groups in pre-ranked GSEA. Furtherrmore, we found that host defense immunity (leukocyte fraction, lymphocyte infiltration, and macrophage fractions) was decreased in primary tumors with Late recurrence compared with Survivors. Utilizing the CIBERSORT algorithm, we showed anti-cancer lymphocytes, memory CD4+ T cells and γδT cells, were significantly lower, and pro-cancerous regulatory T cells were significantly higher in Late tumors compared with Survivors. In agreement, cytolytic activity score that assesses immune cell cytolytic activity was significantly lower in Late compared with Survivors. We demonstrated that not only host defense immunity, but also pro-cancerous immune cells and immune cell cytolytic activity in primary BC was associated with late recurrence.

## Introduction

Nearly all breast cancer (BC)-related deaths are caused by recurrent and/or metastatic breast cancer, rather than the primary tumor. The majority of BC metastasis does not appear at the same time as the primary tumor and the time to recurrence varies considerably. Late recurrence, which occurs five or more years after the initial primary diagnosis, indicates a long dormant period of undetectable metastases, which therefore presents a significant clinical challenge for BC. Accurate and reliable estimates of the risk of late recurrence would enable appropriate management. Thus, there have been a number of attempts reported to predict the timing of BC recurrence. For instance, tumor size and lymph node metastasis have repeatedly been shown to be associated with late recurrence^1–4^; however, many argue that the accuracy of these clinical parameters are insufficient predictors to appropriately guide management^5^. Roughly 20–40% of estrogen receptor (ER) + BC patients eventually develop distant metastasis, and half of these events occur five or more years after diagnosis of the primary tumor^6^. This is in sharp contrast to ER-negative tumors, for which the recurrence rate peaks at around two years, but the rate diminishes after five years^7^. There have been attempts to utilize multi-parametric molecular assays, such as IHC4, OncotypeDX, EndoPredict, PAM50 risk of recurrence score, and Breast Cancer Index, to predict late recurrence in addition to early recurrence (relapse less than five years after initial treatment)^1,8^. However, many of these markers are not specifically tailored to predict late recurrence, as some are reportedly predictive of not only early but also late recurrence. While gene expression signatures that are retrospectively associated with late recurrences have recently been identified by comparing the gene expression profiles of primary tumors of early vs. late recurrences^6^, or using dormant cancer cells in experimental systems^9^, it remains to be determined whether these signatures can prospectively predict late recurrence.

Given the limitations described above, accurately risk-stratifying primary tumors as to their propensity for late recurrence remains a major clinical challenge in BC. Tumor infiltrating lymphocytes (TILs) are immune cells that have migrated to the tumor tissue and the local microenvironment^10^. The presence of TILs in tumor tissue is a result of the immune response generated by the patient against the malignancy. Recently, evidence has emerged demonstrating the importance of TILs in breast cancer as follows: the presence of TILs has been shown to correlate with a good prognosis and higher rates of pathological complete response to neoadjuvant chemotherapy^10^. Host factors are suggested to influence the timing of cancer recurrence since the processes and factors that have been implicated in dormancy include angiogenesis^11,12^, immune-surveillance^13–15^, and a wide variety of microenvironment cues such as extracellular matrix, growth factors and cytokines. Therefore, TILs may also be greatly involved in the timing of breast cancer recurrence.

We hypothesize that the host’s immune status may be closely related to the timing of cancer recurrence. We examined the relationship between the timing of cancer recurrence and clinical factors, gene expression profiles, and immune status utilizing collected data from The Cancer Genome Atlas (TCGA) and Molecular Taxonomy of Breast Cancer International Consortium (METABRIC) primary BC cohorts.

## Materials and Methods

### Data acquisition

TCGA was supervised by the National Cancer Institute (NCI) and the National Human Genome Research Institute^16^. The gene expression levels (mRNA expression z-score from RNA-sequence) from Genomic Identification of Significant Targets in Cancer for TCGA cohort was downloaded through cBioportal (TCGA provisional dataset)^17,18^. The values of “progression free survival (PFI)” and “PFI time” were obtained from (Liu *et al*., 2018 dataset)^19^. We defined timing of cancer recurrence as Early; recurrence ≤2 years, **Mid**; recurrence between 2–5 years, Late; recurrence >5 years, and Survivors; no recurrence >5 years. In the TCGA BC cohort, out of 934 primary BC patients, 308 women, excluding 626 women without relapse but not followed for 5 years, were analyzed. Out of a total of 308 women with recurrence or follow up data in the TCGA BC cohort, one hundred and twenty-three (39.9%) BC patients developed recurrent tumors, 49 Early, 54 Mid, 20 Late, and 185 BC patients were Survivors. The Nottingham Grade was calculated based on tubule formation, nuclear pleomorphism, and mitotic count, which were obtained from the TIE database containing pathology reports of the TCGA BC cohort patients. The gene expression levels (mRNA expression z-score from microarray) from METABRIC cohort was downloaded through cBioportal (METABRIC Nature 2012 & Nat Commun 2016 dataset). The values of relapse status (distant and local) and their relapse time were used as obtained from (Rueda *et al*., 2019 dataset)^20^. Out of 1,904 primary BC patients in METABRIC, 1,727 primary BC were used for distant and local recurrence analysis except for 274 women without distant and local recurrence but not followed for 5 years and 1,410 primary BC were used for breast cancer specific death (BSD) analysis except for 494 women alive but not followed for 10 years. They were used to support the authenticity of the association between timing of cancer recurrence and gene expression and TILs^21,22^. In recurrence analysis, out of a total of 1,727 women with BC, 582 (35.7%) patients developed distant recurrent tumors, 170 Early, 213 Mid, 199 Late, and 92 (8.8%) BC patients developed local recurrent tumors alone, 19 Early, 21 Mid, and 57 Late, and 1,048 BC patients were Survivors.

### Statistical analyses of RNA expression and loneliness

The analysis followed a two-step process. First, we calculated the fold changes of genes, corresponding to each timeframe of cancer recurrence (whole, Early, Mid, and Late), which provided a list of *t*-scores and corresponding *p*-values for each timeframe of cancer recurrence in relation to each of the gene expression values. Second, gene set enrichment analysis was performed in Gene Set Enrichment Analyses (GSEA) Pre-ranked using these collections of gene sets from the Hallmarks gene sets using software provided by the Broad Institute (http://software.broadinstitute.org/gsea/index.jsp). We only considered gene sets significantly enriched that met a threshold of normalized enrichment score (NES) >1.5 or <−1.5 and false discovery rate (FDR) q-value < 0.01.

### Immune characteristics analysis

We used a previously developed dataset^23^ to examine the association between timing of cancer recurrence and immune characteristics (intratumoral immune states, antigen-specific T cell receptor (TCR) and B cell receptor (BCR) repertoires, and immune subtypes). These previously defined “intratumoral immune states” were characterized using scores of 160 immune expression signatures and cluster analysis to identify modules of immune signature sets. “Immune subtypes” were defined as follows: C1 (wound healing) had elevated expression of angiogenic genes, a high proliferation rate, and a Th2 cell bias to the adaptive immune infiltrate, which was related with luminal A BC. C2 (IFN-γ dominant) had the highest M1/M2 macrophage polarization, a strong CD8 signal and, together with C6, the greatest TCR diversity. C2 also showed a high proliferation rate, which may override an evolving type I immune response, and was comprised of highly mutated BC. C3 (inflammatory) was defined by elevated Th17 and Th1 genes, low to moderate tumor cell proliferation, and, along with C5, lower levels of aneuploidy and overall somatic copy number alterations than the other subtypes. C4 (lymphocyte depleted) displayed a more prominent macrophage signature with Th1 suppressed and a high M2 response. C5 (immunologically quiet) exhibited the lowest lymphocyte and highest macrophage responses, dominated by M2 macrophages. C6 (TGF-β dominant) displayed the highest TGF-β signature and a high lymphocytic infiltrate with an even distribution of type I and type II T cells.

To evaluate intra-tumor immune cell composition, the relative fraction of 22 immune cell types in tumor tissue was estimated using the CIBERSORT deconvolution algorithm^24^, as described before^25^. These 22 cell fractions were calculated via the online calculator (https://cibersort.stanford.edu/) as previously shown^25^. The immune cytolytic activity (CYT) was defined as the geometric mean of GZMA and PRF1 expression values in Transcripts Per Million (TPM). The gene expression data were obtained in RSEM format from the Genomic Data Common data and converted to TPM by a given gene’s estimated fraction of transcripts and multiplying with 10^6^26,27^. CYT was calculated as previously described^25^.

### Statistical analysis

All statistical analyses were performed using R software (http:///www.r-project.org/) and Bioconductor (http://bioconductor.org/). The chi-square test or Fisher’s exact test or the nonparametric Mann-Whitney U test and contingency analysis were used to assess baseline differences between binary variables. The Kruskal–Wallis test was used to assess the relationship between mRNA expression and timing of cancer recurrence. Correlations were calculated using Spearman’s rank correlation coefficient. In the analysis of disease free survival (DFS), the Kaplan–Meier method was used to estimate survival rates, and differences between survival curves were evaluated by the log-rank test. Cox’s proportional hazards model was used for the univariate and multivariate analysis of prognostic status. Two-sided *P* values < 0.05 was considered as statistically significant for all tests.

## Results

### Association between clinical features of the primary tumors and the timing of cancer recurrence

We studied the relationship between clinical features of the primary tumor and the timing of cancer recurrence in TCGA BC cohort (Table 1) and METABRIC cohort (Tables 2 and 3). Compared with Survivors without recurrence, the primary tumor which developed Early recurrence was significantly associated with a larger tumor size (p = 0.0061), lymph node metastasis (p = 0.037), higher Nottingham Grade (p < 0.0001), higher clinical stage (p < 0.0001), negative ER (p = 0.0085), and negative progesterone receptor (PgR) (p = 0.0023) in TCGA BC cohort (Table 1). In addition to all the above mentioned features, positive human epidermal growth receptor 2 (HER2) (p < 0.00001), low frequency of the hormone receptor (HR) + HER2− group (p < 0.00001), no treatment with adjuvant endocrine therapy (p = 0.045), and treatment with adjuvant chemotherapy (p < 0.00001) were associated with Early in distant metastasis analysis of METABRIC cohort. Compared to Survivors, Mid-term recurrence was significantly associated with lymph node metastasis (p = 0.00086) and higher clinical stage (p = 0.00093) in TCGA. In METABRIC, Mid was significantly associated with older age (p = 0.0075) and postmenopausal status (p = 0.0077), as well as clinical features significantly associated with the Early group. Interestingly, there was no statistically significant difference in clinical features between Survivors and Late recurrence group in TCGA, whereas, Late was significantly associated with lymph node metastasis (p = 0.000029), positive ER (p = 0.014), high frequency of the HR + HER2− group (p = 0.0017), and treatment with adjuvant endocrine therapy (p = 0.014), compared to Survivors in distant metastasis analysis of METABRIC (Table 2). In the local recurrence analysis of METABRIC cohort, Late was significantly associated with age (p = 0.035), premenopausal status (p = 0.035), positive PgR (p = 0.049), and treatment with radiation therapy (p = 0.021), compared to Survivors. Interestingly, there was no statistically significant difference in clinical characteristics between Early and Late and Survivors in the local recurrence analysis (Table 3). In addition, although we verified the relationship between timing of BSD and clinical features in METABRIC cohort, the results were similar to those of the TCGA BC cohort and the distant metastasis analysis in METABRIC cohort (Table S1). These results indicate that primary tumors that develop Late recurrence, particularly, local recurrence, were not as clinically aggressive as Early and Mid recurrence, and had almost the same features as Survivors.

**Table 1:** Patients and clinical characteristics associated with cancer recurrence timeframe in TCGA cohort.

Variables		Number of Patients (%)						
		Survivors	Recurrence					
			Early	*P*-value (vs Survivors)	Mid	*P*-value (vs Survivors)	Late	*P*-value (vs Survivors)
		(*N* = 185)	(*N* = 49)		(*N* = 54)		(*N* = 20)	
Age	50≥	63 (34.1)	14 (28.6)	0.47	21 (38.9)	0.51	11 (55)	0.063
	50<	122 (65.9)	35 (71.4)		33 (61.1)		9 (45)	
Race	Caucasian American	148 (80)	35 (71.4)	0.39	40 (74.1)	0.86	15 (75)	NA
	African American	29 (15.7)	9 (18.4)		9 (16.7)		4 (20)	
	Asian	5 (2.7)	3 (6.1)		2 (3.7)		0	
	Unknown	3 (1.6)	2 (4.1)		3 (5.6)		1 (5)	
Menopausal state	Pre	49 (26.5)	10 (20.4)	0.24	16 (29.6)	0.57	5 (25)	0.26
	Post	105 (56.8)	34 (69.4)		28 (51.9)		5 (25)	
	Unknown	31 (16.8)	5 (10.2)		10 (18.5)		10 (50)	
Tumor size (cm)	2≥	157 (84.9)	32 (65.3)	0.0061*	41 (75.9)	0.23	17 (85)	0.83
	2 <	32 (17.3)	17 (34.7)		13 (24.1)		3 (15)	
Lymphnode	Negative	99 (53.5)	18 (36.7)	0.037*	15 (27.8)	0.00086*	8 (40)	0.25
	Positive	86 (46.5)	31 (63.3)		39 (72.2)		12 (60)	
Histopathology	Ductal	141 (76.2)	37 (75.5)	0.51	39 (72.2)	0.62	16 (80)	0.35
	Lobular	36 (19.5)	7 (14.3)		12 (22.2)		2 (10)	
	Others/unknown	8 (4.3)	5 (10.2)		3 (5.6)		2 (10)	
Nottingham Grade	1/2	56 (30.3)	7 (14.3)	0.018*	12 (22.2)	0.22	1 (5)	0.74
	3	35 (18.9)	14 (28.6)		13 (24.1)		1 (5)	
	unknown	94 (50.8)	28 (57.1)		29 (53.7)		18 (90)	
Clinical stage	I/II	150 (81.1)	24 (49)	<0.0001*	32 (59.3)	0.00093*	15 (75)	0.51
	III/IV	35 (18.9)	25 (51)		22 (40.7)		5 (25)	
ER	Negative	46 (24.9)	21 (42.9)	0.0085*	18 (33.3)	0.15	2 (10)	0.13
	Positive	137 (74.1)	26 (53.1)		33 (61.1)		18 (90)	
	Unknown	2 (1.1)	2 (4.1)		3 (5.6)		0	
PgR	Negative	64 (34.6)	28 (57.1)	0.0023*	22 (40.7)	0.25	5 (25)	0.36
	Positive	118 (63.8)	19 (38.8)		28 (51.9)		15 (75)	
	unknown	2 (1.1)	2 (4.1)		3 (5.6)		0	
HER2	Negative	138 (74.6)	37 (75.5)	0.79	37 (68.5)	0.25	5 (25)	0.3
	Positive	23 (12.4)	7 (14.3)		3 (5.6)		2 (10)	
	Unknown	24 (13.0)	5 (10.2)		14 (25.9)		13 (65)	
Subtype	HR+ ^a^HER2−	106 (57.3)	22 (44.9)	0.11	23 (42.6)	0.096	5 (25)	NA
	HER2+	23 (12.4)	7 (14.3)		3 (5.6)		2 (10)	
	TN^b^	32 (17.3)	15 (30.6)		14 (25.9)		0	
	Unknown	24 (13.0)	5 (10.2)		14 (25.9)		13 (65)	

Abbreviations: TCGA, The Cancer Genome Atlas; ER, estrogen receptor; PgR, progesterone receptor; HER2, human epidermal growth factor receptor 2; HR, hormone receptor; TN, triple; NA, not available.

^a^HR+: ER-positive and/or PgR-positive.

^b^TN: HR-negative and HER2-negative.

*Factor showing statistical significance. The chi-square test and Fisher’s extract test were used to assess baseline differences between binary variables. P < 0.05 is considered statistically significant.

**Table 2:** Patients and clinical characteristics associated with timing of distant recurrence in the METABRIC cohort.

Variables		Number of Patients (%)						
		Survivors	Recurrence					
			Early	*P*-value (vs Survivors)	Mid	*P*-value (vs Survivors)	Late	*P*-value (vs Survivors)
		(*N* = 1048)	(*N* = 170)		(*N* = 213)		(*N* = 199)	
Age	50≥	209 (19.9)	45 (26.5)	0.051	60 (28.2)	0.0075*	40 (20.1)	0.99
	50<	839 (80.1)	125 (73.5)		153 (71.8)		159 (79.9)	
Menopausal state	Pre	209 (19.9)	45 (26.5)	0.053	60 (28.2)	0.0077*	40 (20.1)	0.96
	Post	838 (80)	125 (73.5)		153 (71.8)		159 (79.9)	
	Unknown	1 (0.1)	0		0		0	
Tumor size (cm)	2≥	513 (49)	49 (28.8)	<0.00001*	66 (31)	<0.00001*	86 (43.2)	0.12
	2 <	524 (50)	119 (70)		146 (68.5)		112 (56.3)	
	Unknown	11 (1)	2 (1.2)		1 (0.5)		1 (0.5)	
Lymphnode	Negative	636 (60.7)	52 (30.6)	<0.00001*	81 (38)	<0.00001*	89 (44.7)	0.000029*
	Positive	412 (39.3)	118 (69.4)		132 (62)		110 (55.3)	
Histopathology	Ductal	783 (74.7)	148 (87.1)	0.28	165 (77.5)	0.87	147 (73.9)	0.9
	Lobular	77 (7.3)	10 (5.9)		17 (8)		15 (7.5)	
	Others/unknown	188 (17.9)	12 (7.1)		31 (14.6)		37 (18.6)	
Tumor grade	1/2	542 (51.7)	44 (25.9)	<0.00001*	75 (35.2)	<0.00001*	112 (56.3)	0.24
	3	466 (44.5)	122 (71.9)		132 (62)		80 (40.2)	
	unknown	40 (3.8)	4 (2.4)		6 (2.8)		7 (3.5)	
Clinical Stage	I/II	723 (69)	86 (50.6)	<0.00001*	137 (64.3)	<0.00001*	140 (70.4)	0.096
	III/IV	35 (3.3)	31 (18.2)		27 (12.7)		12 (6)	
	Unknown	290 (27.7)	53 (31.2)		49 (23)		47 (23.6)	
ER	Negative	220 (21)	82 (48.2)	<0.00001*	65 (30.5)	0.0024*	19 (9.5)	0.00017*
	Positive	828 (79)	88 (51.8)		148 (69.5)		180 (90.5)	
PgR	Negative	455 (43.4)	126 (74.1)	<0.00001*	114 (53.5)	0.0071*	77 (38.7)	0.21
	Positive	592 (56.5)	44 (25.9)		99 (46.5)		122 (61.3)	
	Unknown	1 (0.1)	0		0		0	
HER2	Negative	947 (90.4)	133 (78.2)	<0.00001*	163 (76.5)	<0.00001*	181 (91)	0.82
	Positive	100 (9.5)	37 (21.8)		50 (23.5)		18 (9)	
	Unknown	1 (0.1)	0		0		0	
Subtype	HR+ ^a^HER2−	781 (74.5)	80 (47.1)	<0.00001*	125 (58.7)	<0.00001*	168 (84.4)	0.0022*
	HER2+	100 (9.5)	37 (21.8)		50 (23.5)		18 (9)	
	TN^b^	166 (15.8)	53 (31.2)		38 (17.8)		13 (6.5)	
	Unknown	1 (0.1)	0		0		0	
Molecular Characterization	Luminal A	419 (40)	23 (13.5)	<0.00001*	45 (21.1)	<0.00001*	77 (38.7)	0.0017*
	Luminal B	224 (21.4)	36 (21.2)		79 (37.1)		58 (29.1)	
	HER2	96 (9.2)	34 (20)		38 (17.8)		24 (12.1)	
	Basal-like	104 (9.9)	45 (26.5)		26 (12.2)		6 (3)	
	Claudin-low	124 (11.8)	20 (11.8)		11 (5.2)		16 (8)	
	Normal	77 (7.3)	11 (6.5)		13 (6.1)		17 (8.5)	
	Unknown	4 (0.3)	1 (0.6)		1 (0.5)		1 (0.5)	
Radiation therapy	No	418 (39.9)	56 (32.9)	0.83	79 (37.1)	0.44	86 (43.2)	0.39
	Yes	629 (60)	114 (67.1)		134 (62.9)		113 (56.8)	
	Unknown	1 (0.1)	0		0		0	
Adjuvant Endocrine therapy	No	396 (37.8)	78 (45.9)	0.045*	90 (42.3)	0.22	57 (28.6)	0.014*
	Yes	652 (62.2)	92 (54.1)		123 (57.7)		142 (71.4)	
Adjuvant chemotherapy	No	872 (83.2)	95 (55.9)	<0.00001*	145 (68.1)	<0.00001*	164 (82.4)	0.76
	Yes	175 (16.7)	75 (44.1)		68 (31.9)		35 (17.6)	
	Unknown	1 (0.1)	0		0		0	

Abbreviations: METABRIC, Molecular Taxonomy of Breast Cancer International Consortium; ER, estrogen receptor; PgR, progesterone receptor; HER2, human epidermal growth factor receptor 2; HR, hormone receptor; TN, triple; NA, not available.

^a^HR+: ER-positive and/or PgR-positive.

^b^TN: HR-negative and HER2-negative.

*Factor showing statistical significance. The chi-square test and Fisher’s extract test were used to assess baseline differences between binary variables. P < 0.05 is considered statistically significant.

**Table 3:** Patients and clinical characteristics associated with the timing of local recurrence in the METABRIC cohort.

Variables		Number of Patients (%)						
		Survivors	Recurrence					
			Early	*P*-value (vs Survivors)	Mid	*P*-value (vs Survivors)	Late	*P*-value (vs Survivors)
		(*N* = 1048)	(*N* = 19)		(*N* = 21)		(*N* = 57)	
Age	50≥	209 (19.9)	6 (31.6)	0.21	5 (23.8)	0.66	18 (31.6)	0.035*
	50<	839 (80.1)	13 (68.4)		16 (76.2)		39 (68.4)	
Menopausal state	Pre	209 (19.9)	6 (31.6)	0.21	5 (23.8)	0.66	18 (31.6)	0.035*
	Post	838 (80)	13 (68.4)		16 (76.2)		39 (68.4)	
	Unknown	1 (0.1)	0		0		0	
Tumor size (cm)	2≥	513 (49)	9 (47.4)	0.86	11 (52.4)	0.79	30 (52.6)	0.46
	2<	524 (50)	10 (52.6)		10 (47.6)		25 (43.9)	
	Unknown	11 (1)	0		0		2 (3.5)	
Lymphnode	Negative	636 (60.7)	14 (73.7)	0.25	13 (61.9)	0.91	29 (50.9)	0.14
	Positive	412 (39.3)	5 (26.3)		8 (38.1)		28 (49.1)	
Histopathology	Ductal	783 (74.7)	14 (73.7)	0.054	14 (66.7)	0.21	47 (82.5)	0.24
	Lobular	77 (7.3)	4 (21.1)		3 (14.3)		2 (3.5)	
	Others/unknown	188 (17.9)	1 (5.3)		4 (19)		8 (14)	
Tumor grade	1/2	542 (51.7)	10 (52.6)	0.92	12 (57.1)	0.28	28 (49.1)	0.99
	3	466 (44.5)	9 (47.4)		6 (28.6)		24 (42.1)	
	unknown	40 (3.8)	0		3 (14.3)		5 (8.8)	
Clinical Stage	I/II	723 (69)	14 (73.7)	0.71	16 (76.2)	0.81	43 (75.4)	0.55
	III/IV	35 (3.3)	1 (5.3)		1 (4.8)		3 (5.3)	
	Unknown	290 (27.7)	4 (21.1)		4 (19)		11 (19.3)	
ER	Negative	220 (21)	4 (21.1)	0.99	1 (4.8)	0.069	7 (12.3)	0.11
	Positive	828 (79)	15 (78.9)		20 (95.2)		50 (87.7)	
PgR	Negative	455 (43.4)	10 (52.6)	0.42	5 (23.8)	0.071	14 (24.6)	0.049*
	Positive	592 (56.5)	9 (47.4)		16 (76.2)		43 (75.4)	
	Unknown	1 (0.1)	0		0		0	
HER2	Negative	947 (90.4)	17 (89.5)	0.89	19 (90.5)	1	54 (94.7)	0.28
	Positive	100 (9.5)	2 (10.5)		2 (9.5)		3 (5.3)	
	Unknown	1 (0.1)	0		0		0	
Subtype	HR+ ^a^HER2−	781 (74.5)	13 (68.4)	0.81	19 (90.5)	—	47 (82.5)	0.38
	HER2+	100 (9.5)	2 (10.5)		2 (9.5)		3 (5.3)	
	TN^b^	166 (15.8)	4 (21.1)		0		7 (12.3)	
	Unknown	1 (0.1)	0		0		0	
Molecular Characterization	Luminal A	419 (40)	11 (57.9)	—	13 (61.9)	—	27 (47.4)	0.3
	Luminal B	224 (21.4)	4 (21.1)		2 (9.5)		14 (24.6)	
	HER2	96 (9.2)	0		2 (9.5)		5 (8.8)	
	Basal-like	104 (9.9)	3 (15.8)		0		1 (1.8)	
	Claudin-low	124 (11.8)	1 (5.3)		0		6 (10.5)	
	Normal	77 (7.3)	0		4 (19)		4 (7)	
	Unknown	4 (0.3)	0		0		0	
Radiation therapy	No	418 (39.9)	10 (52.6)	0.26	11 (52.4)	0.25	14 (24.6)	0.021*
	Yes	629 (60)	9 (47.4)		10 (47.6)		43 (75.4)	
	Unknown	1 (0.1)	0		0		0	
Adjuvant Endocrine therapy	No	396 (37.8)	9 (47.4)	0.39	8 (38.1)	0.98	23 (40.4)	0.7
	Yes	652 (62.2)	10 (52.6)		13 (61.9)		34 (59.6)	
Adjuvant chemotherapy	No	872 (83.2)	18 (94.7)	0.18	17 (81)	0.78	46 (80.7)	0.61
	Yes	175 (16.7)	1 (5.3)		4 (19)		11 (19.3)	
	Unknown	1 (0.1)	0		0		0	

Abbreviations: METABRIC, Molecular Taxonomy of Breast Cancer International Consortium; ER, estrogen receptor; PgR, progesterone receptor; HER2, human epidermal growth factor receptor 2; HR, hormone receptor; TN, triple; NA, not available.

^a^HR+: ER-positive and/or PgR-positive.

^b^TN: HR-negative and HER2-negative.

*Factor showing statistical significance. The chi-square test and Fisher’s extract test were used to assess baseline differences between binary variables. P < 0.05 is considered statistically significant.

### Gene expression differences in early, mid, and late recurrence

**Figure 1:**
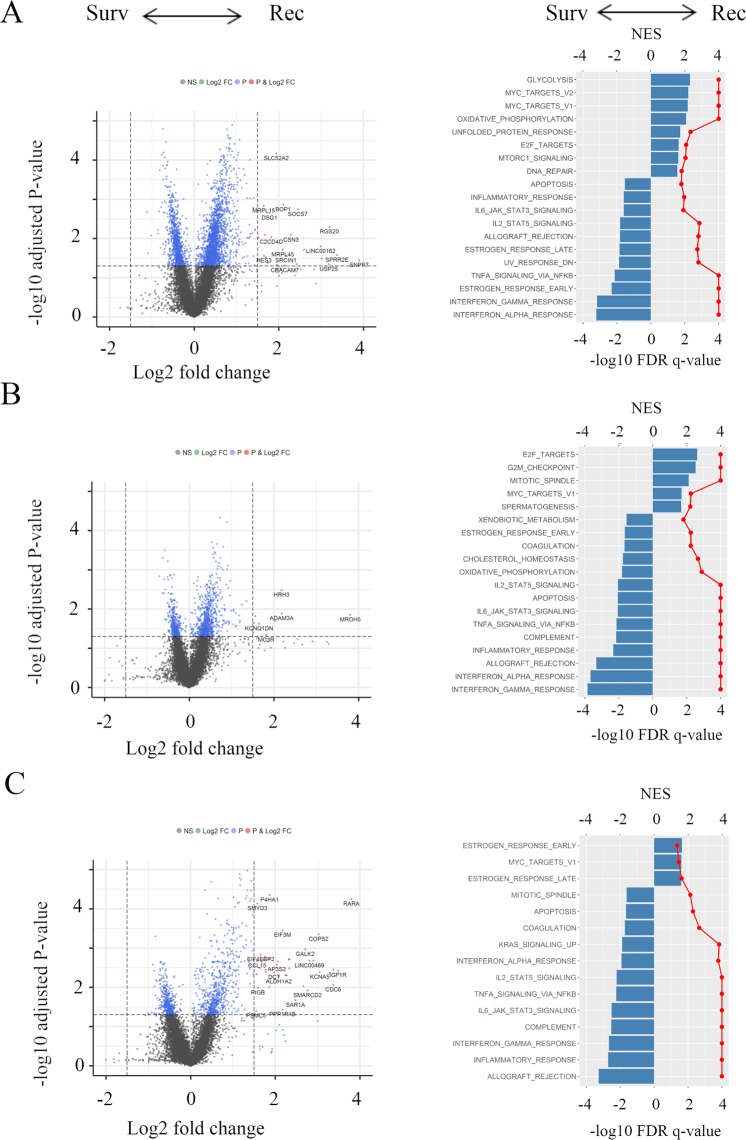
Volcano plots illustrating the differentially expressed mRNAs of BC and pre-ranked GSEA of BC patients comparing Survivors and Early recurrence. (**A)** Mid recurrence; (**B)** and Late recurrence; (**C)** in TCGA BC cohort. Primary BCs with cancer recurrence data were analyzed and categorized as follows: recurrence ≤2 years (**Early**), recurrence between 2–5 years (**Mid**), recurrence >5 years (**Late**), and no recurrence >5 years (**Survivors**). Left panels: In volcano plots, X-axes: log2 FC; Y-axes: −log 10 P-value from limma analysis. mRNAs with P-value < 0.05 and FC >1.5 are marked in red, with P-value < 0.05 and FC <1/1.5 in green, all others in black. Right panels: In pre-ranked GSEA, blue bar shows NES and red dots show –log10 FDR q-value. We only considered gene sets significantly enriched that met a threshold of NES >1.5 or <−1.5 and FDR q-value < 0.01. Abbreviations: BC, breast cancer; GESA, Gene Set Enrichment Analyses; TCGA, The Cancer Genome Atlas; FC, fold change; NES, normalized enrichment score; FDR, false discovery rate.

In order to clarify the mechanisms associated with the timing of cancer recurrence, volcano plots and gene set enrichment assays were performed comparing that with Survivors. Volcano plots, representing the distribution of the fold changes and adjusted p-values of 18,428 genes, and the Hallmark gene sets in pre-ranked GSEA were shown in Fig. 1 corresponded to the timing of cancer recurrence in the TCGA BC cohort. mRNA in recurrent versus non-recurrent breast tumors revealed 28 mRNAs in Early, 12 mRNAs in Mid, and 45 mRNAs in Late which were differentially expressed with fold change greater than 1.5 and p < 0.05. Interestingly, all detected genes were up-regulated in the recurrence groups. In pre-ranked GSEA, in the Early group, Glycolysis (NES = 2.31, FDR *q* < 0.0001) and MYC target gene sets (V1; NES = 2.18, FDR *q* < 0.0001, V2; NES = 2.21, FDR *q* < 0.0001) were enriched compared with the Survivors group (Fig. 1A). In the Mid group, cell cycle related gene sets (E2F targets; NES = 2.62, FDR *q* < 0.0001, G2M checkpoint; NES = 2.53, FDR *q* < 0.0001, Mitotic Spindle; NES = 2.11, FDR *q* < 0.0001) were enriched (Fig. 1B). However, in the Late group, estrogen response gene sets (early; NES = 1.64, FDR *q* = 0.0043 and late; NES = 1.60, FDR *q* = 0.0024) and MYC target v1 (NES = 1.60, FDR *q* = 0.034)) were enriched (Fig. 1C). Interestingly, the Survivors group enriched interferon (IFN)-α/-γ response and TNF-α signaling via NFκβ gene sets in all groups.

**Figure 2:**
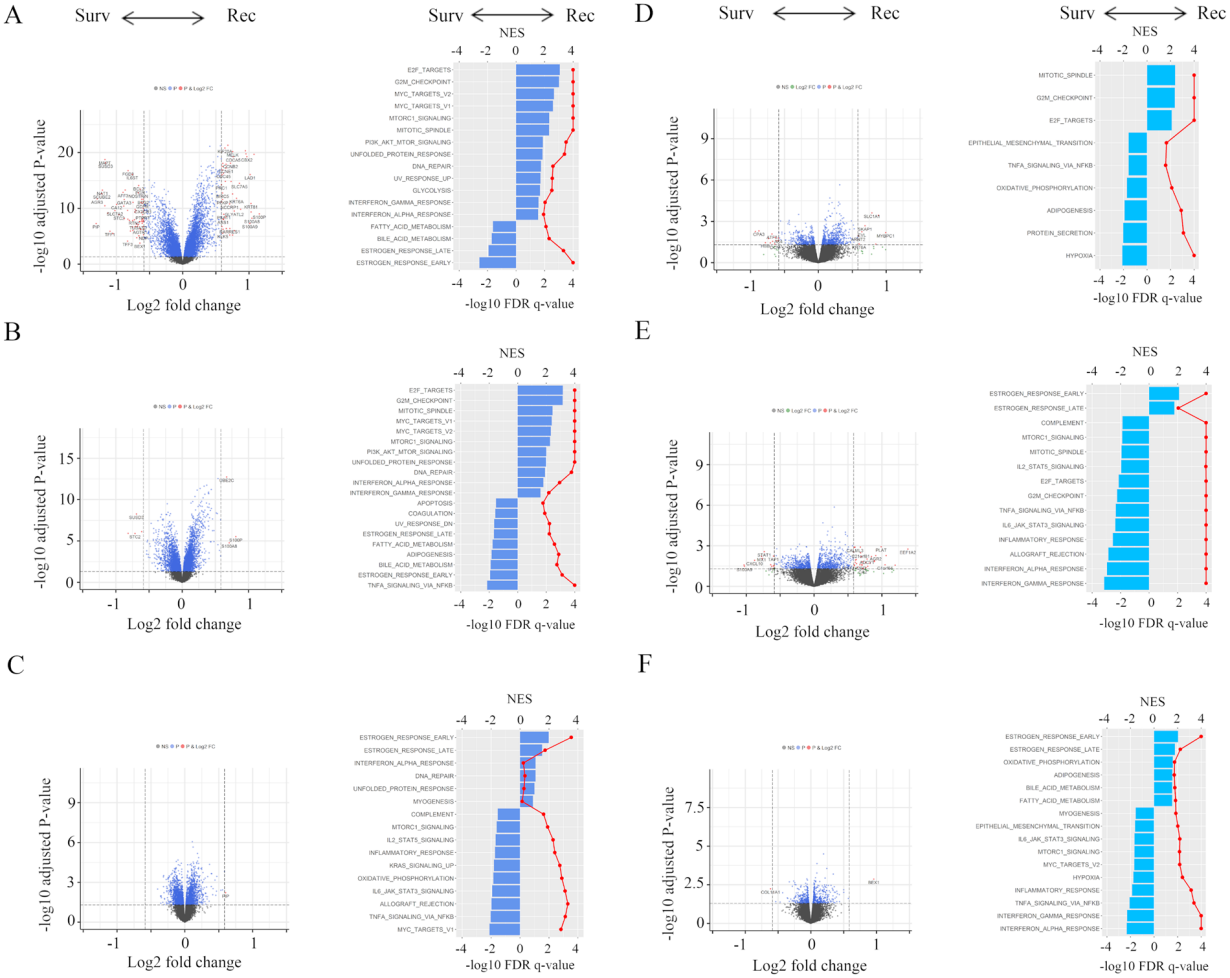
Volcano plots illustrating the differentially expressed mRNAs of BC and pre-ranked GSEA of BC patients comparing Survivors and Early distant recurrence. (**A)** Mid distant recurrence; (**B)** and Late distant recurrence; (**C)** and comparing Survivors and Early local recurrence; (**D)** Mid local recurrence; (**E)** and Late local recurrence; (**F)** in METABRIC cohort. Primary BCs with cancer recurrence data were analyzed and categorized as follows: recurrence ≤2 years (**Early**), recurrence between 2–5 years (**Mid**), recurrence >5 years (**Late**), and no recurrence >5 years (**Survivors**). Left panels: In volcano plots, X-axes: log2 FC; Y-axes: −log 10 P-value from limma analysis. mRNAs with P-value < 0.05 and FC >log2(1.5) are marked in red, with P-value < 0.05 and FC <log2(1/1.5) in green, all others in black. Right panels: In pre-ranked GSEA, blue bar shows NES and red dots show –log10 FDR q-value. We only considered gene sets significantly enriched that met a threshold of NES >1.5 or <−1.5 and FDR q-value < 0.01. Abbreviations: BC, breast cancer; GESA, Gene Set Enrichment Analyses; METABRIC, Molecular Taxonomy of Breast Cancer International Consortium; FC, fold change; NES, normalized enrichment score; FDR, false discovery rate.

Figure 2 shows Volcano plots, representing the distribution of the fold changes and adjusted p-values of 18,484 genes, and the Hallmark gene sets in pre-ranked GSEA, corresponding to timing of cancer recurrence in the METABRIC cohort. mRNA in each recurrence timeframe versus Survivors revealed that, in distant metastasis analysis, 115 significant mature mRNAs in Early, in which 47 mRNAs (40.9%) were up-regulated and 68 (59.1%) were downregulated, 7 significant mature mRNAs in Mid, in which 3 mRNAs (42.9%) were up-regulated and 4 (57.1%) were downregulated, 1 mRNA significant up-regulated mRNA in Late (A–C), and, in local recurrence analysis, 36 significant mature mRNAs in Early, in which 17 mRNAs (47.2%) was up-regulated and 19 mRNAs (52.8%) were down-regulated, 72 significant mature mRNAs in Mid, in which 55 mRNAs (76.4%) were up-regulated and 17 (23.6%) were downregulated, 2 significant mature mRNAs in Late, in which 1 mRNAs were up-regulated and 1 were downregulated (D–F), all of which were differentially expressed with fold change greater than log2(1.5) and p < 0.05. In pre-ranked GSEA, in distant metastasis analysis, cell cycle related gene sets (E2F targets; NES = 3.07, FDR *q* < 0.0001, G2M checkpoint; NES = 3.01, FDR *q* < 0.0001, Mitotic Spindle; NES = 2.32, FDR *q* < 0.0001), MYC target gene sets (V1; NES = 2.59, FDR *q* < 0.0001, V2; NES = 2.66, FDR *q* < 0.0001), and mTORC1 signaling (NES = 2.32, FDR *q* < 0.0001) were enriched in the Early group (Fig. 2A). Similarly, cell cycle related gene sets (E2F targets; NES = 3.17, FDR *q* < 0.0001, G2M checkpoint; NES = 3.16, FDR *q* < 0.0001, Mitotic Spindle; NES = 2.44, FDR *q* < 0.0001), MYC target gene sets (V1; NES = 2.39, FDR *q* < 0.0001, V2; NES = 2.33, FDR *q* < 0.0001), mTORC1 signaling (NES = 2.27, FDR *q* < 0.0001), and PI3K AKT mTOR signaling (NES = 2.01, FDR *q* < 0.0001) were enriched in the Mid group (Fig. 2B). In the Late group, estrogen response gene sets (early; NES = 2.00, FDR *q* < 0.0001 and late; NES = 1.53, FDR *q* = 0.018) were enriched (Fig. 2C). Interestingly, Survivors enriched MYC targets v1 (NES = −2.11, FDR *q* = 0.01) as well as TNF-α signaling via NFκβ (NES = −2.02, FDR *q* = 0.01) compared to that of the Late group (Fig. 2C). In local recurrence analysis, cell cycle related gene sets (E2F targets; NES = 2.11, FDR *q* < 0.0001, G2M checkpoint; NES = 2.37, FDR *q* < 0.0001, Mitotic Spindle; NES = 2.40, FDR *q* < 0.0001) were enriched in the Early group (Fig. 2D). Interestingly, early and late estrogen response gene sets were both enriched in the Mid group (early; NES = 2.12, FDR *q* < 0.0001 and late; NES = 1.78, FDR *q* = 0.009). In the Late group, estrogen response gene sets (early; NES = 2.04, FDR *q* < 0.0001 and late; NES = 1.78, FDR *q* = 0.006) were enriched and Survivors correlated with IFN-α/-γ response and TNF-α signaling via NFκβ gene sets in the Mid and the Late group (Fig. 2F). In agreement with the results of recurrence analysis in TCGA and METABRIC, Early BSD significantly enriched cell-cycle related gene sets, MYC targets, and mTORC1 signaling, Mid BSD significantly enriched cell-cycle related gene sets, Late BSD significantly enriched Estrogen Response, and Survivors which TNF-α signaling via NFκβ and IFN-γ response were significantly enriched (Fig. S1). These results indicated that Late recurrence was associated with estrogen response compared as Survivors as described previously^6^. More interestingly, Survivors were associated with TNF-α signaling via NFκβ compared with recurrence groups.

### Tumor immune microenvironment differs by cancer recurrences timeframe

**Figure 3:**
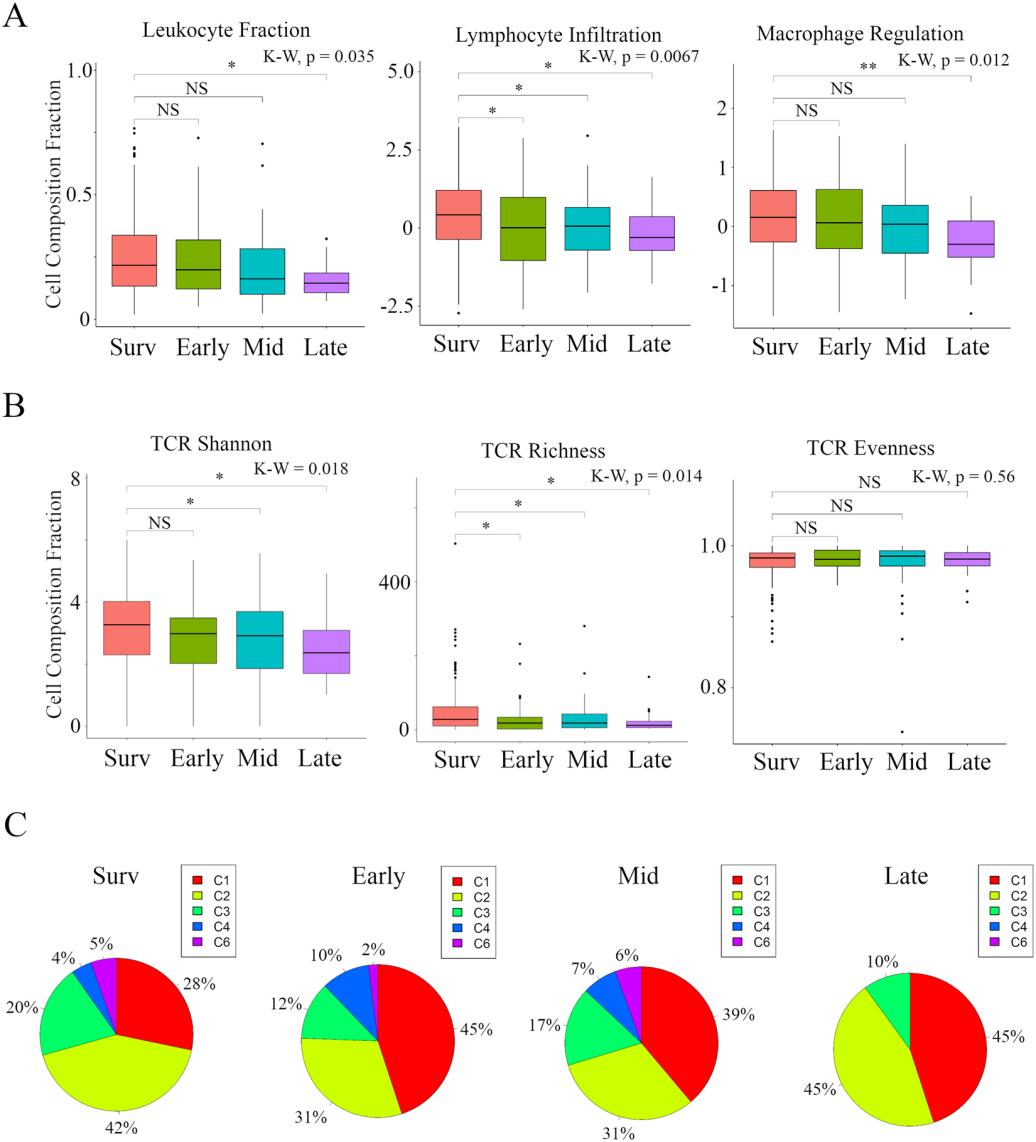
Tumor immune microenvironment differs by timings of breast cancer recurrence in TCGA BC cohort. Box plots of the relationship between each timeframe of cancer recurrence and immune cell fractions (left to right; Leukocyte Fraction, and Lymphocyte Infiltration, and Macrophage Regulation); (**A)** and TCR repertoire (left to right; Shannon, Richness, and Evenness). (**B**,**C)** Pie chart of “Immune Subtypes” in each cancer recurrence timeframe. Primary BCs with cancer recurrence data was analyzed and categorized as follows: recurrence ≤2 years (**Early**), recurrence between 2–5 years (**Mid**), recurrence >5 years (**Late**), and no recurrence >5 years (**Survivors**). ***Means P < 0.001, **means P < 0.01 and *means P < 0.05. Abbreviations: TCGA, The Cancer Genome Atlas; BC, breast cancer; TCR, T cell receptor; K-W, Kruskal-Wallis; NS, not significant.

To assess the tumor immune microenvironment, leukocyte fraction, lymphocyte infiltration, macrophage regulation, antigen-specific TCR and BCR, and previously defined “Immune Subtypes”^23^ were compared among the primary tumors by the timing of recurrence. Five immune expression signatures were composed of macrophages/monocytes, overall lymphocyte infiltration (dominated by T and B cells), TGF-β response, IFN-γ response, and wound healing, which robustly reproduced co-clustering of these immune signature sets^23^. Interestingly, both leukocyte fraction and macrophage regulation were significantly lower only in the Late group, whereas lymphocyte infiltration was statistically significantly lower in all the tumors that recurred regardless of timing (Early, Mid, and Late), indicating that weak host defense cancer immunity correlated with recurrence, particularly in Late (Fig. 3A). Antigen-specific TCR and BCR repertoires are critical for the recognition of pathogens and malignant cells and may reflect a robust anti-tumor response comprising a large number of antigen specific adaptive immune cells that have undergone clonal expansion and effector differentiation^23^. We demonstrated the relationship between TCR and BCR repertoires and timing of cancer recurrence in Fig. 3B. Lower TCR diversity was associated with later recurrence (Mid and Late recurrence in Shannon Entropy and all recurrence in Richness), but there was no correlation between BCR repertoire and timing of cancer recurrence. The six resulting clusters “Immune Subtypes”, C1–C6, were characterized using a distinct distribution of scores over the above five immune expression signatures^23^. We described the relationship between these “Immune Subtypes” and timing of cancer recurrence in Fig. 3C. As a matter of course, we did not identify C5 (immunologically quiet). Although it was not statistically significant, Late was associated with C1 (wound healing) and C2 (IFN-γ dominant), but it was only slightly associated with C3 (inflammatory) and it was not associated with C4 (lymphocyte depleted) or C6 (TGF-β dominant). These results indicate that host defense immunity, including leukocyte fraction, lymphocyte infiltration, macrophage regulation, and TCR diversity, was suppressed in the Late recurrence group compared with Survivors.

### Breast cancer recurrence was associated with low Tumor-Infiltrating Lymphocytes (TILs), high Tumor Associated Macrophages, and low immune cytolytic activity (CYT)

**Figure 4:**
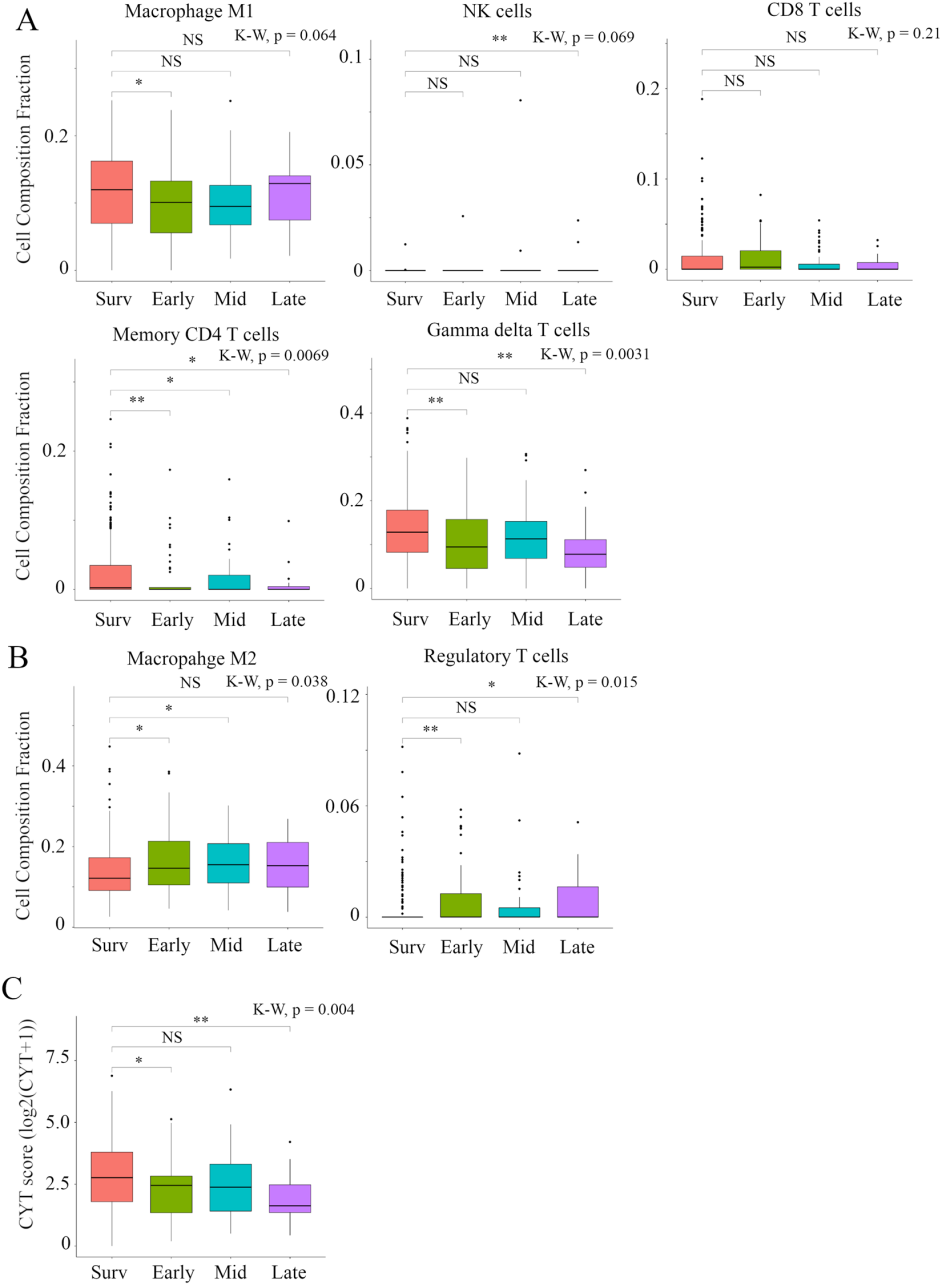
Box plots of immune cell components and CYT score comparison between timing of cancer recurrence in TCGA BC cohort. (**A**) Anti-cancer immune cells, (**B)** pro-cancerous immune cells, and **C**, CYT scores were shown. Primary BCs with cancer recurrence data were analyzed and categorized as follows: recurrence ≤2 years (**Early**), recurrence between 2–5 years (**Mid**), recurrence >5 years (**Late**), and no recurrence >5 years (**Survivors**). **Means P < 0.01 and *means P < 0.05. Abbreviations: CYT, immune cytolytic activity; TCGA, The Cancer Genome Atlas; BC, breast cancer; K-W, Kruskal-Wallis; NS, not significant.

**Figure 5:**
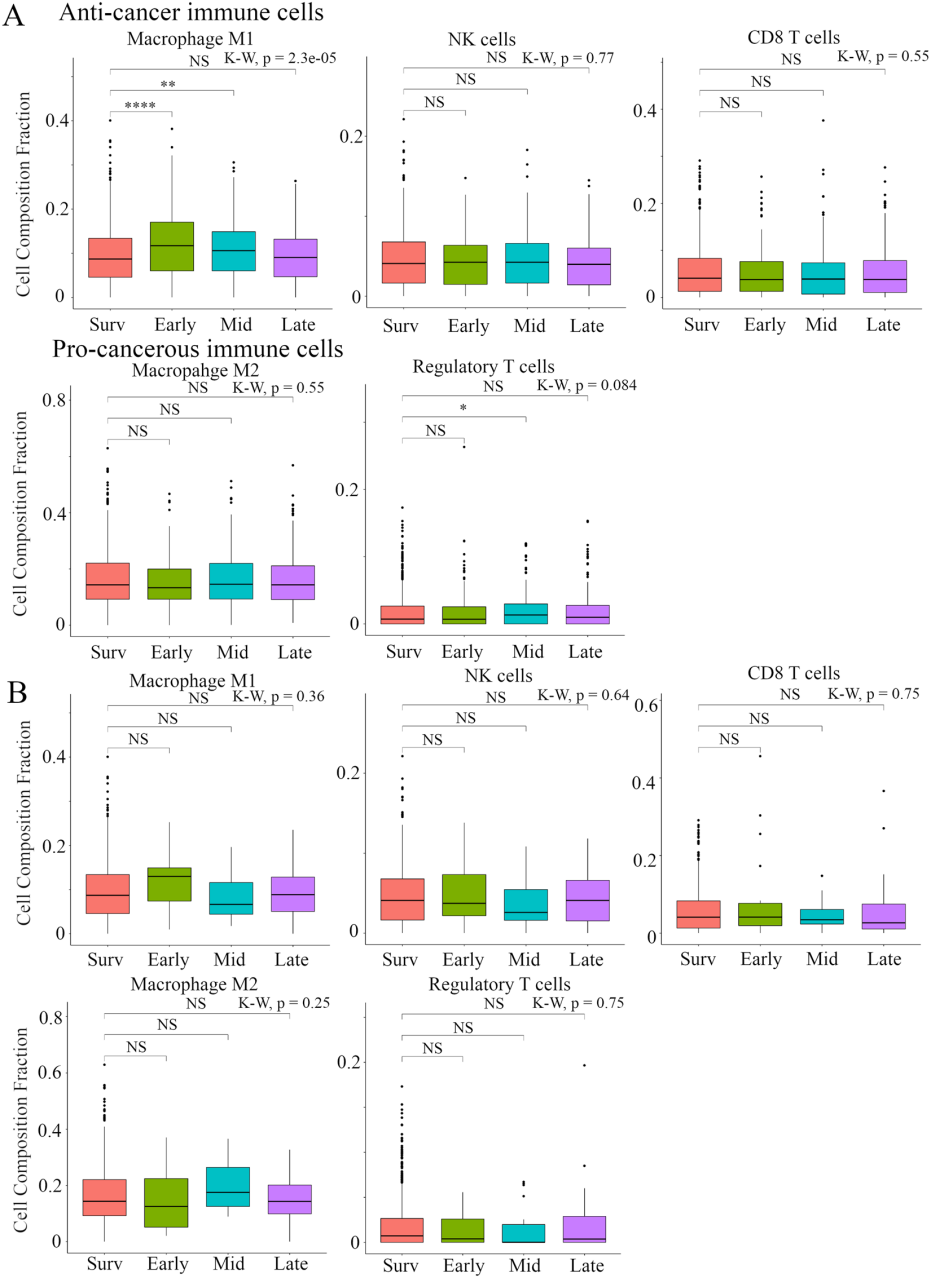
Box plots of immune cell components comparison between timing of BC recurrence in METABRIC cohort. Anti-cancer immune cells (upper) and pro-cancerous immune cells (bottom) were shown in distant recurrence section, (**A)** and in local recurrence section. (**B)** Primary BCs with cancer recurrence data were analyzed and categorized as follows: recurrence ≤2 years (**Early**), recurrence between 2–5 years (**Mid**), recurrence >5 years (**Late**), and no recurrence >5 years (**Survivors**). ****Means P < 0.0001, **means P < 0.01 and *means P < 0.05. Abbreviations: BC, breast cancer; METABRIC, Molecular Taxonomy of Breast Cancer International Consortium;.K-W, Kruskal-Wallis; NS, not significant.

In order evaluate the tumor immune microenvironment in recurrent tumors, we analyzed the immune cell composition utilizing CIBERSORT and/or CYT in the TCGA BC cohort (Fig. 4**)** and METABRIC cohort (Fig. 5). In TCGA BC cohort, we found that anti-cancer M1 macrophages were lower in Early, while pro-cancerous M2 macrophages were higher in Early and Mid compared to Survivors. Anti-cancer activated memory CD4+ T cells were significantly lower in all recurrence groups, and anti-cancer γδT cells were significantly lower and pro-cancerous regulatory T cells were significantly higher in Early and Late compared to Survivors. It is well established that CYT scores represent anti-cancer immune activity and the killing of malignant cells by TILs^26^. Accordingly, CYT score was significantly lower in Early and Late compared to Survivors. In the METABRIC cohort, in distant recurrence analysis, we found that anti-cancer M1 macrophages were higher in Early and Mid compared to Survivors. Pro-cancerous regulatory T cells were significantly higher in Mid compared to Survivors. Interestingly, in local recurrence analysis, there was no statistically significant difference between timing of cancer recurrence and Survivors (Fig. 5). In agreement with the results of recurrence analysis in TCGA and METABRIC, anti-cancer M1 macrophages were higher in Early BSD and pro-cancerous M2 macrophages were higher in Mid BSD compared to Survivors. Furthermore, anti-cancer resting memory CD4+ T cells were significantly lower in Early and Mid BSD and pro-cancerous regulatory T cells were significantly higher in Mid and Late BSD compared to Survivors (Fig. S2). These results indicated that Late recurrence was associated with pro-cancerous immune compositions and low cytolytic activity of immune cells compared to Survivors.

### Low CYT in primary tumors was associated with late recurrence in the TCGA BC cohort

**Figure 6:**
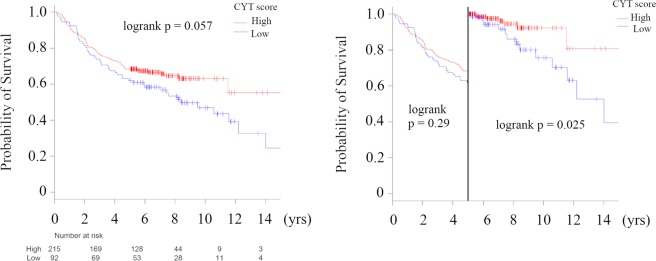
Kaplan-Meier plots of the association of the presence of CYT score with DFS in TCGA BC cohort; the whole cohort; Left Panel, Early + Mid vs Late; Right Panel. High CYT score was defined as ≥30^th^ percentile of CYT score. Primary BCs with cancer recurrence data were analyzed and categorized as follows: recurrence ≤2 years (**Early**), recurrence between 2–5 years (**Mid**), recurrence >5 years (**Late**), and no recurrence >5 years (**Survivors**). **Means P < 0.01 and *means P < 0.05. Abbreviations: CYT, immune cytolytic activity; DFS, disease-specific survival; TCGA, The Cancer Genome Atlas; BC, breast cancer; K-W, Kruskal-Wallis; NS, not significant.

In order to verify that low CY T can serve as a predictive biomarker of Late recurrence, we examined the relationship between CYT and the whole cohort and earlier (Early + Mid) and Late recurrence (Fig. 6). Patients with low CYT were marginally associated with worse DFS (*p* = 0.057), which were tested by the Kaplan–Meier method and verified by the log-rank (Mantel–Cox) test. Next, we examined the relationship between low CYT and DFS by timing of cancer recurrence. CYT was not associated with DFS in Early, but it was significantly associated with worse DFS in Late (p = 0.025). The DFS Cox hazard analysis for timing of cancer recurrence is shown in Table S2. The results showed that low CYT score was a significantly worse prognostic parameter in Late (univariate analysis; hazard ratio (HR): 0.36, 95% confidence interval (CI): 0.14–0.91, *p* = 0.031, multivariate analysis; HR: 0.29, 95% CI: 0.11–0.76, *p* = 0.012), but not in Early (univariate analysis; HR: 0.8, 95%CI: 0.83–1.88, *p* = 0.28, multivariate analysis; HR: 0.7, 95% CI: 0.93–2.16, *p* = 0.1). Interestingly, in the Late group, clinical factors, such as tumor size, node metastasis, and clinical stage, were not correlated with prognosis. These results indicated that immune cell cytolytic activity was a relevant prognostic factor for late recurrence.

## Discussion

As late recurrence in BC remains a challenge despite advances in overall BC survival, studies have focused on efforts to more accurately and reliably predict the risk of late BC recurrence. While prior studies have shown the importance of clinical factors^1–5^, subtypes^6,7^, and gene signatures^1,8^, the relationship between late recurrence and immune status has yet to be demonstrated. Accordingly, we showed that BC patient who develop recurrence earlier (Early and Mid) had primary tumors associated with more aggressive clinical characteristics such as larger tumor, more lymph node metastases, higher pathological grades, higher Stages, and negative ER and PgR, compared to Survivors; however, clinical characteristics of primary tumors with Late recurrence were almost the same as Survivors (Tables 1–3). In addition, we showed that a decrease in host defense immunity, activation of pro-cancerous immune cells and a decrease in immune cell cytolytic activity in BC were closely related to late recurrence by computational biologically analyzing two large primary BC cohorts. This study generated three interesting results with clinical implications. First, primary tumors of Survivors were associated with anti-cancer signaling such as INF-α/-γ response and TNF-α signaling, compared with the recurrence groups (Figs 1 and S1). In addition, in both distant and local recurrence analyses, Survivors correlated with TNF-α signaling via NFκβ compared to the Late group (Fig. 2). These results support the hypothesis that immune system status is implicated in the prevention of BC recurrence^28^. Furthermore, primary tumors with earlier recurrence (Early and Mid) were mainly associated with cell cycle related gene sets and MYC target gene sets involved in BC exacerbation and primary tumors with Late recurrence were associated with estrogen signaling, compared with Survivors, as described previously^1–8^ (Figs 1 and S1). Interestingly, in local recurrence, estrogen response gene sets were found to be more predominant than those of distant metastasis. Second, host defense immunity (leukocyte fraction, lymphocyte infiltration, and macrophage fractions) was decreased in primary tumors with Late recurrence compared with Survivors. In addition, primary tumors with Late recurrence were significantly associated with low diversity of TCR and specific “Immune Subtypes”, such as, C1 (wound healing) and C2 (IFN-γ dominant) (Fig. 3). To our knowledge, there has been no report that host defense immunity is involved in BC late recurrence. Finally, late recurrence was associated with activation of pro-cancerous immune cells and a decrease in cytolytic activity of immune cells in primary breast tumors. Utilizing the CIBERSORT algorithm, we showed that anti-cancer lymphocytes, memory CD4+ T cells and γδT cells, were significantly lower, and pro-cancerous regulatory T cells were significantly higher in Late tumors compared to Survivors (Fig. 4). In agreement, CYT score that assesses immune cell cytolytic activity was significantly lower in primary tumors with Late recurrence compared to Survivors and low CYT score in primary tumors was statistically significantly associated with worse DFS in the Late group (Figs 4 and 6). Interestingly, in local recurrence, there was no statistically significant difference between timing of cancer recurrence and Survivors (Fig. 5). It has been reported that BCs are infiltrated with diverse populations of immune system cells and these infiltrates appear to be associated with disease outcome^6^. For example, patients with gene signatures of Th1/CTL phenotype were shown to have favorable outcomes whereas Th2/B-cell related genes were more likely to occur in patients with HR−/HER2− disease^29^. In addition, some translational studies in patients with breast carcinoma have suggested that infiltration by pro-cancerous immune cells such as regulatory T cells might have a great response to chemotherapy and might affect the clinical outcome^10^. However, there were no reports as we have shown that pro-cancerous immune cells in tumor tissue may be involved in the timing and type of recurrence of breast cancer.

In general, late recurrence seems to be a reflection of a very slowly proliferation of BC cells dormant in distant sites^6^. The fact that dormant micrometastases stay in distant organs for many years suggests a long evolutionary process of these cells after their departure from the primary tumor. During this time, independent genetic and epigenetic traits may arise and drive the recurrences which will not be present in the original primary tumors^30^. However, we did not access the gene expression and distribution of immune cells in recurrence tumors by timing of cancer recurrence. The methods of assessing immune infiltrates in BC are quite varied and due to these differences individual studies are not comparable to each other. Liquid biopsy, which is a non-invasively conducted genetic test using genes extracted from body fluids such as blood and urine, has been developed as a way of providing relevant predictive information related to the tumor tissue as previously demonstrated^29,31–34^. If tumor immune microenvironment can be monitored by liquid biopsy, it is expected to deepen the understanding of the authentic clinical and prognostic value of immune system cells in BC patients.

Although the study demonstrates promising results, it has limitations. First, this is a retrospective study utilizing publicly available datasets, thus it is prone to selection bias. Second, this study is based on the gene expression of the primary tumor in TCGA and METABRIC cohorts, and as it does not include any *in vitro* or *in vivo* experiments it also therefore does not delve deeply into the mechanism of our results to further understand the correlations reported.

In conclusion, we demonstrated the relationship between late recurrence and clinical factors, gene expression profiles, and immune status utilizing collected data from TCGA and METABRIC primary BC cohorts. Not only host defense immunity, but also pro-cancerous immune cells and cytolytic activity of immune cells were associated with Late recurrence in primary BC. Based on these reported results, we anticipate that further research can be conducted to establish a greater understanding of the role of immune cells in BC cancer recurrence.

## Supplementary information

Supplementary file
