# Supplementary material for: Late recurrence of breast cancer is associated with pro-cancerous immune microenvironment in the primary tumor
Source: Sci Rep. 2019 Nov 15;9:16942. doi: 10.1038/s41598-019-53482-x (PMC6858361; doi:10.1038/s41598-019-53482-x)
Supplement: Supplementary file 1 — Supplementary file [file 41598_2019_53482_MOESM1_ESM.docx]

**Supplementary files**

Late recurrence of breast cancer is associated with pro-cancerous immune microenvironment in the primary tumor

Takashi Takeshita^1^, Li Yan^2^, Mariko Asaoka^1^, Omar Rashid^3-5^, and Kazuaki Takabe^1,6-10^

^1^Breast Surgery, Department of Surgical Oncology, Roswell Park Comprehensive Cancer Center, Buffalo, NY, USA

^2^Department of Biostatistics and Bioinformatics, Roswell Park Comprehensive Cancer Center, Buffalo, NY, USA.

^3^ Holy Cross Hospital, Trinity Health, Fort Lauderdale, FL, USA

^4^ Massachusetts General Hospital, Boston, MA, USA

^5^ University of Miami Miller School of Medicine, Miami, FL, USA

^6^Department of Surgery, University at Buffalo Jacobs School of Medicine and Biomedical Sciences, the State University of New York, Buffalo ,NY

^7^Department of Breast Surgery and Oncology, Tokyo Medical University, Tokyo, Japan

^8^Department of Surgery, Yokohama City University, Yokohama, Japan

^9^Department of Surgery, Niigata University Graduate School of Medical and Dental Sciences, Niigata, Japan

^10^Department of Breast Surgery, Fukushima Medical University, Fukushima, Japan

**Figures**

**Fig. S1** Volcano plots illustrating the differentially expressed mRNAs of BC and pre-ranked GSEA of BC patients comparing Survivors and whole BSD; **A**, Early BSD; **B**, Mid BSD; **C**, and Late BSD; **D** in METABRIC cohort. Primary BCs in METABRIC with BSD data was divided into; death ≤5 years (**Early**), death between 5-10 years (**Mid**), death >10 years (**Late**), and survived >10 years (**Survivors**). Out of a total of 1410 women with BC, 622 (44.1%) patients developed BSD, 325 Early, 174 Mid, 123 Late BSD, and 788 patients were Survivors. **Left panels:** In volcano plots, X-axes: log2 FC; Y-axes: -log 10 P-value from limma analysis. mRNAs with P-value < 0.05 and FC > log2(1.5) are marked in red, with P-value < 0.05 and FC < log2(1/1.5) in green, all others in black. 3 significant mature mRNAs in the total, in which 1 was up-regulated and 2 were down-regulated, 65 significant mature mRNAs in Early, in which 21 mRNAs (32.3%) were up-regulated and 44 (67.7%) were downregulated, 8 significant mature mRNAs in Mid, in which one mRNAs (12.5%) were up-regulated and 7 (87.5%) were downregulated, but there was not significant mature mRNA in Late BSD, all of which were differentially expressed with fold change greater than log2(1.5) and p < 0.05. **Right panels:** In pre-ranked GSEA, blue bar shows NES and red dots show –log10 FDR q-value. We only considered gene sets significantly enriched that met a threshold of NES >1.5 or <-1.5 and FDR q-value < 0.01. Whole BSD significantly enriched cell-cycle related gene sets (G2M checkpoint; NES=3.25, FDR *q*<0.0001, E2F targets; NES=3.26, FDR *q*<0.0001, and mitotic spindle; NES=2.47, FDR *q*<0.0001), MYC targets (v1;NES=2.39, FDR *q*<0.0001, and v2;NES=2.58 , FDR *q*<0.0001), and MTORC1 signaling; NES=2.47, FDR *q*<0.0001, but it did not significantly correlated with Estrogen response (early; NES=-2.43, FDR *q*<0.0001, and late; NES=-1.90, FDR *q*=0.0016). Early BSD significantly enriched cell-cycle related gene sets (G2M checkpoint; NES=3.15, FDR *q*<0.0001, E2F targets; NES=3.27, FDR *q*<0.0001, and mitotic spindle; NES=2.16, FDR *q*<0.0001), MYC targets (v1;NES=2.71, FDR *q*<0.0001, and v2;NES=2.63 , FDR *q*<0.0001), and MTORC1 signaling; NES=2.63, FDR *q*<0.0001, but it did not significantly correlated with Estrogen response (early; NES=-2.59, FDR *q*<0.0001, and late; NES=-2.00, FDR *q*<0.0001). Mid BSD significantly enriched cell-cycle related gene sets (G2M checkpoint; NES=2.21, FDR *q*<0.0001, E2F targets; NES=2.12, FDR *q*<0.0001, but it did not significantly correlated with TNF-α signaling via NFκβ (NES=-2.20, FDR *q*<0.0001) and KRAS signaling up (NES=-2.11, FDR *q*<0.0001). Late BSD significantly enriched Estrogen response (early; NES=2.17, FDR *q*<0.0001 and late; NES=1.78, FDR *q*=0.0033), but it did not significantly correlated with TNF-α signaling via NFκβ (NES=-2.42, FDR *q*<0.0001) and IFN-γ response (NES=-2.33, FDR *q*<0.0001).

**Abbreviations**: BC, breast cancer; GESA, Gene Set Enrichment Analyses; BSD, breast cancer specific death; METABRIC, Molecular Taxonomy of Breast Cancer International Consortium; FC, fold change; NES, normalized enrichment score; FDR, false discovery rate; IFN, interferon.


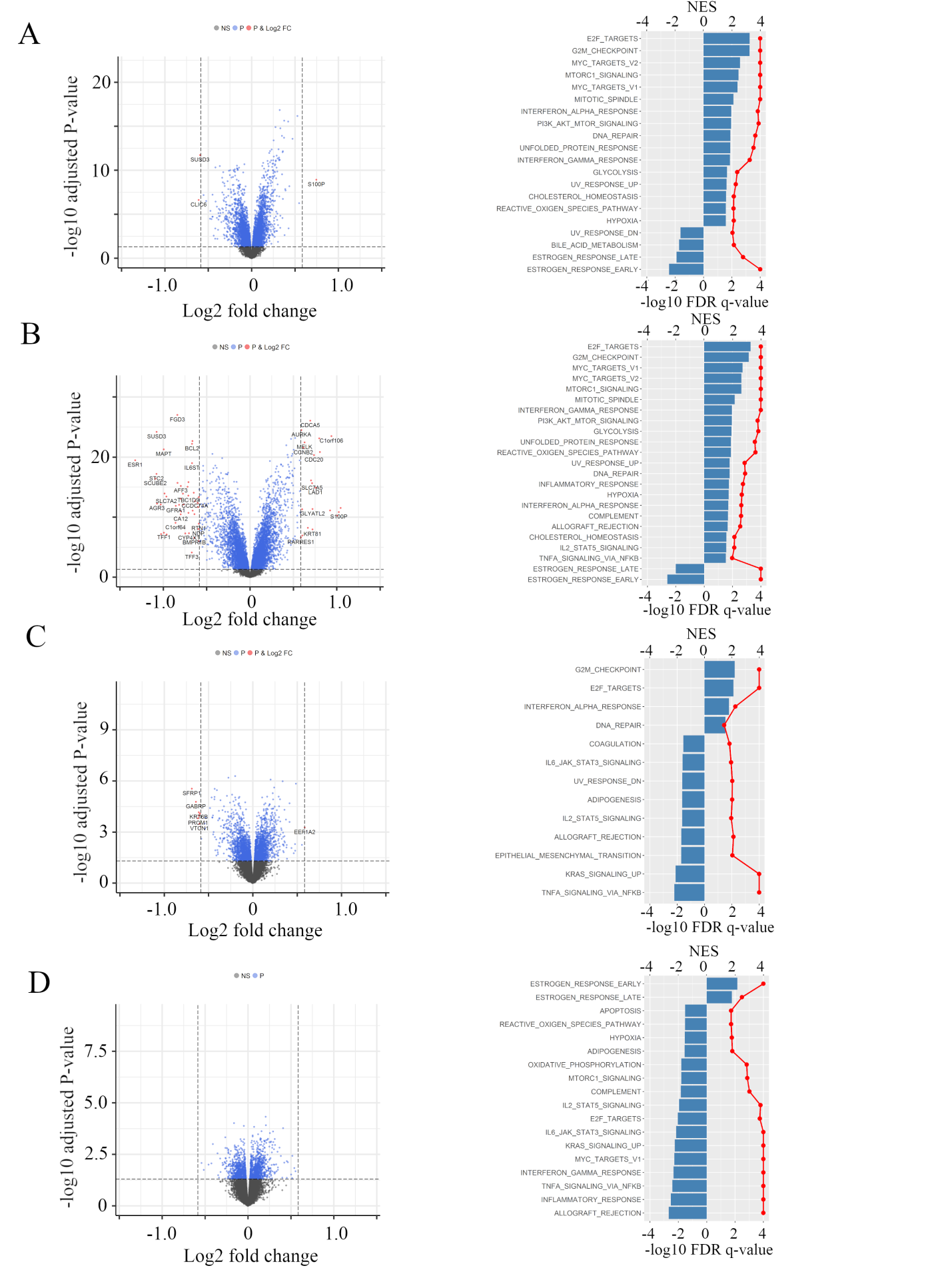


**Fig. S2** Box plots of immune cell components comparison between timing of BC recurrence in METABRIC cohort. Anti-cancer immune cells (upper) and pro-cancerous immune cells (bottom) were shown. Primary BCs in METABRIC with BSD data was divided into; death ≤5 years (**Early**), death between 5-10 years (**Mid**), death >10 years (**Late**), and survived >10 years (**Survivors**). *** means P < 0.001, ** means P<0.01 and * means P < 0.05.

**Abbreviations**: BC, breast cancer; METABRIC, Molecular Taxonomy of Breast Cancer International Consortium; BSD, breast cancer specific death; K-W, Kruskal-Wallis; NS, not significant.


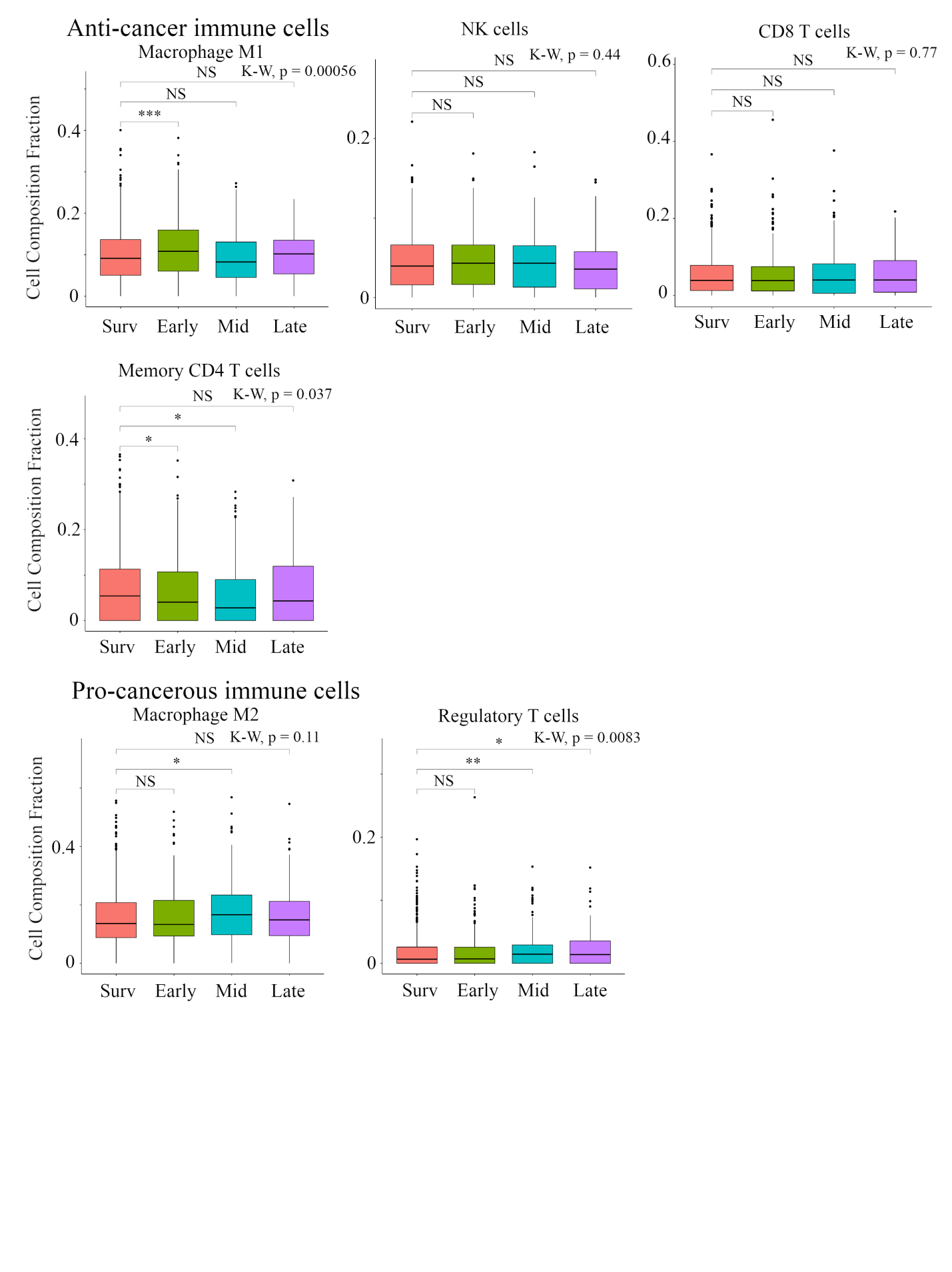


**Tables**

**Table S1**: Patients and clinical characteristics associated with timing of BSD in METABRIC cohort

| Variables | | Number of Patients (%) | | | | | | |
| --- | --- | --- | --- | --- | --- | --- | --- | --- |
|  |  | Survivors | BCS death | | | | | |
|  |  |  | Early | *P*-value (vs Survivors) | Mid | *P*-value (vs Survivors) | Late | *P*-value (vs Survivors) |
|  |  | (*N* = 788) | (*N* = 325) |  | (*N* = 174) |  | (*N* = 123) |  |
| Age | 50 > | 188 (23.9) | 92 (28.3) | 0.12 | 28 (16.1) | 0.026* | 31 (25.2) | 0.75 |
|  | 50 < | 600 (76.1) | 233 (71.7) |  | 146 (83.9) |  | 92 (74.8) |  |
| Menopausal state | Pre | 188 (23.9) | 92 (28.3) | 0.12 | 28 (16.1) | 0.026* | 31 (25.2) | 0.75 |
|  | Post | 599 (76.0) | 233 (71.7) |  | 146 (83.9) |  | 92 (74.8) |  |
|  | Unknown | 1 (0.1) | 0 |  | 0 |  | 0 |  |
| Tumor size (cm) | 2 > | 412 (52.3) | 87 (26.8) | < 0.0001* | 71 (40.8) | 0.0037* | 48 (39.0) | 0.0052* |
|  | 2 < | 366 (46.4) | 234 (72) |  | 103 (59.2) |  | 74 (60.2) |  |
|  | Unknown | 10 (1.3) | 4 (1.2) |  | 0 |  | 1 (0.8) |  |
| Lymphnode | Negative | 483 (61.3) | 94 (28.9) | < 0.0001* | 75 (43.1) | < 0.0001* | 65 (52.8) | 0.075 |
|  | Positive | 305 (38.7) | 231 (71.1) |  | 99 (56.9) |  | 58 (47.2) |  |
| Histopathology | Ductal | 596 (75.6) | 266 (81.8) | 0.87 | 139 (79.9) | 0.99 | 87 (70.7) | 0.8 |
|  | Lobular | 56 (7.1) | 24 (7.4) |  | 13 (7.5) |  | 9 (7.3) |  |
|  | Others/unknown | 136 (17.3) | 35 (10.8) |  | 22 (12.6) |  | 27 (22.0) |  |
| Tumor grade | 1/2 | 409 (51.9) | 87 (26.8) | < 0.0001* | 83 (47.7) | 0.22 | 65 (52.8) | 0.95 |
|  | 3 | 344 (43.7) | 231 (71.1) |  | 86 (49.4) |  | 54 (43.9) |  |
|  | unknown | 35 (4.4) | 7 (2.2) |  | 5 (2.9) |  | 4 (3.3) |  |
| Clinical Stage | I/II | 556 (70.6) | 180 (55.4) | < 0.0001* | 125 (71.8) | < 0.0001* | 89 (72.4) | < 0.0001* |
|  | III/IV | 2 (0.3) | 56 (17.2) |  | 13 (7.5) |  | 6 (4.9) |  |
|  | Unknown | 204 (25.9) | 89 (27.4) |  | 36 (20.7) |  | 28 (22.8) |  |
| ER | Negative | 164 (20.8) | 151 (46.5) | < 0.0001* | 21 (12.1) | 0.0081* | 14 (11.4) | 0.014* |
|  | Positive | 624 (79.2) | 174 (53.5) |  | 153 (87.9) |  | 109 (88.6) |  |
| PgR | Negative | 336 (42.6) | 222 (68.3) | < 0.0001* | 80 (46) | 0.43 | 40 (32.5) | 0.033* |
|  | Positive | 451 (57.2) | 103 (31.7) |  | 94 (54) |  | 83 (67.5) |  |
|  | Unknown | 1 (0.1) | 0 |  | 0 |  | 0 |  |
| HER2 | Negative | 716 (90.9) | 249 (76.6) | < 0.0001* | 144 (82.8) | 0.0014* | 113 (91.9) | 0.75 |
|  | Positive | 71 (9.0) | 76 (23.4) |  | 30 (17.2) |  | 10 (8.1) |  |
|  | Unknown | 1 (0.1) | 0 |  | 0 |  | 0 |  |
| Subtype | HR+^a^ HER2- | 589 (74.7) | 154 (47.4) | < 0.0001* | 131 (75.3) | 0.00026* | 103 (83.7) | 0.57 |
|  | HER2+ | 71 (9.0) | 76 (23.4) |  | 30 (17.2) |  | 10 (8.1) |  |
|  | TN^b^ | 127 (16.1) | 95 (29.2) |  | 13 (7.5) |  | 10 (8.1) |  |
|  | Unknown | 1 (0.1) | 0 |  | 0 |  | 0 |  |
| Molecular Characterization | Luminal A | 329 (41.8) | 46 (14.2) | < 0.0001* | 51 (29.3) | < 0.0001* | 49 (39.8) | 0.062 |
|  | Luminal B | 164 (20.8) | 84 (25.8) |  | 63 (36.2) |  | 34 (27.6) |  |
|  | HER2 | 71 (9.0) | 69 (21.2) |  | 26 (14.9) |  | 12 (9.8) |  |
|  | Basal-like | 79 (10) | 70 (21.5) |  | 7 (4) |  | 4 (3.3) |  |
|  | Claudin-low | 87 (11) | 34 (10.5) |  | 12 (6.9) |  | 10 (8.1) |  |
|  | Normal | 55 (7.0) | 21 (6.5) |  | 14 (8) |  | 13 (10.6) |  |
|  | Unknown | 3 (0.4) | 1 (0.3) |  | 1 (0.6) |  | 1 (0.8) |  |
| Radiation therapy | No | 307 (39) | 118 (36.3) | 0.4 | 75 (43.1) | 0.32 | 53 (43.1) | 0.74 |
|  | Yes | 480 (60.9) | 207 (63.7) |  | 99 (56.9) |  | 70 (56.9) |  |
|  | Unknown | 1 (0.1) | 0 |  | 0 |  | 0 |  |
| Adjuvant Endocrine therapy | No | 340 (43.1) | 148 (45.5) | 0.46 | 56 (32.2) | 0.0078* | 43 (35) | 0.087 |
|  | Yes | 448 (56.9) | 177 (54.5) |  | 118 (67.8) |  | 80 (65) |  |
| Adjuvant chemotherapy | No | 658 (83.5) | 186 (57.2) | < 0.0001* | 140 (80.5) | 0.32 | 107 (87) | 0.34 |
|  | Yes | 129 (16.4) | 139 (42.8) |  | 34 (19.5) |  | 16 (13) |  |
|  | Unknown | 1 (0.1) | 0 |  | 0 |  | 0 |  |

Abbreviations: BSD, breast cancer specific death; METABRIC, Molecular Taxonomy of Breast Cancer International Consortium; ER, estrogen receptor; PgR, progesterone receptor; HER2, human epidermal growth factor receptor 2; HR, hormone receptor; TN, triple; NA, not available.

^a^ HR+: ER-positive and/or PgR-positive.

^b^ TN: HR-negative and HER2-negative.

* Factor showing statistical significance. The chi-square test and Fisher’s extract test were used to assess baseline differences between binary variables. P < .05 is considered statistically significant

**Table S2**: Univariate and multivariate analysis of recurrence free survival in TCGA cohort

| Early+Mid |  |  |  |  |  |  |  |  |  |  |
| --- | --- | --- | --- | --- | --- | --- | --- | --- | --- | --- |
| Variables | | Univariate analysis | | | |  | Multivariate analysis | | | |
|  |  | Value | HR | 95%CI | *P*-value | n | HR | 95%CI | *P*-value | n |
| CYT score | (ref=30 percentile) | High | 0.8 | 0.83-1.88 | 0.28 | 307 | 0.7 | 0.93-2.16 | 0.1 | 300 |
| ER | (ref=Neg) | Positive | 0.54 | 0.36-0.80 | 0.0026* | 301 | 0.48 | 0.32-0.72 | 0.00039* |  |
| PgR | (ref=Neg) | Positive | 0.54 | 0.36-0.81 | 0.0025* | 299 |  |  |  |  |
| HER2 | (ref=Neg) | Positive | 0.86 | 0.44-1.66 | 0.65 | 252 |  |  |  |  |
| Age | (ref= <50) | 50≥ | 1.12 | 0.74-1.68 | 0.6 | 308 |  |  |  |  |
| Menopausal state | (ref=Post) | Pre | 0.85 | 0.53-1.34 | 0.47 | 252 |  |  |  |  |
| Histopathology | Ductal (ref=Ductal) | Lobular | 1.02 | 0.64-1.62 | 0.93 | 291 |  |  |  |  |
| Tumor size (cm) | (ref=2≥) | >2 | 1.8 | 1.17-2.75 | 0.0069* | 308 | 0.9 | 0.53-1.55 | 0.71 |  |
| Node metastasis | (ref=0) | 1+ | 1.95 | 1.29-2.96 | 1.50E-03* | 308 | 1.19 | 0.69-2.06 | 0.52 |  |
| Clinical stage | (ref= I/II) | III/IV | 2.72 | 1.85-4.02 | 4.45E-07* | 308 | 2.89 | 1.57-5.30 | 6.10E-04* |  |
| Nuclear Grade | (ref=1) | 2/3 | 4.42 | 1.07-18.2 | 0.04* | 139 |  |  |  |  |
|  |  |  |  |  |  |  |  |  |  |  |
| Late |  |  |  |  |  |  |  |  |  |  |
| Variables | | Univariate analysis | | | |  | Multivariate analysis | | | |
|  |  | Value | HR | 95%CI | *P*-value | n | HR | 95%CI | *P*-value | n |
| CYT score | (ref=30 percentile) | High | 0.36 | 0.14-0.91 | 0.031* | 307 | 0.29 | 0.11-0.76 | 0.012* | 307 |
| ER | (ref=Neg) | Positive | 2.5 | 0.58-10.8 | 0.22 | 301 |  |  |  |  |
| PgR | (ref=Neg) | Positive | 1.36 | 0.50-3.84 | 0.53 | 299 |  |  |  |  |
| HER2 | (ref=Neg) | Positive | 3.13 | 0.59-16.5 | 0.18 | 252 |  |  |  |  |
| Age | (ref= <50) | 50≥ | 0.45 | 0.19-1.09 | 0.077 | 308 | 0.35 | 0.14-0.86 | 0.022* |  |
| Menopausal state | (ref=Post) | Pre | 2.62 | 0.73-9.35 | 0.14 | 252 |  |  |  |  |
| Histopathology | Ductal (ref=Ductal) | Lobular | 0.5 | 0.14-1.74 | 0.27 | 291 |  |  |  |  |
| Tumor size (cm) | (ref=2≥) | >2 | 0.61 | 0.18-2.11 | 0.44 | 308 |  |  |  |  |
| Node metastasis | (ref=0) | 1+ | 1.9 | 0.78-4.67 | 0.16 | 308 |  |  |  |  |
| Clinical stage | (ref= I/II) | III/IV | 1.44 | 0.52-3.98 | 0.48 | 308 |  |  |  |  |
| Nuclear Grade | (ref=1) | 2/3 | 8E+07 | 0-Inf | 1 | 139 |  |  |  |  |

Abbreviations: TCGA, The Cancer Genome Atlas; CYT, immune cytolitic activity; ER, estrogen receptor; PgR, progesterone receptor; HER2, human epidermal growth factor receptor 2; HR, hormone receptor; TN, triple; HR, hazard ratio; CI, confidence interval.

* Factor showing statistical significance. The chi-square test and Fisher’s extract test were used to assess baseline differences between binary variables. P < .05 is considered statistically significant
